# Is Acupuncture Effective in Diminishing Frown Lines? Evidence From a Randomized Controlled Trial

**DOI:** 10.1111/jocd.70144

**Published:** 2025-04-07

**Authors:** Hossein Haghir, Mohammad Javad Yazdanpanah, Seyed Kazem Farahmand, Majid Khadem‐Rezaiyan, Hoda Azizi

**Affiliations:** ^1^ Department of Acupuncture, School of Persian and Complementary Medicine Mashhad University of Medical Sciences Mashhad Iran; ^2^ Department of Anatomy and Cell Biology, School of Medicine Mashhad University of Medical Sciences Mashhad Iran; ^3^ Medical Genetic Research Center (MGRC) Mashhad University of Medical Sciences Mashhad Iran; ^4^ Department of Dermatology, School of Medicine Mashhad University of Medical Sciences Mashhad Iran; ^5^ Department of Community Medicine, School of Medicine Mashhad University of Medical Sciences Mashhad Iran

**Keywords:** acupuncture, cosmetic techniques, facial rejuvenation, frown lines, randomized controlled trial, skin aging

## Abstract

**Background:**

As life expectancy rises, facial rejuvenation has gained significance.

**Aims:**

This study aimed to evaluate the effects of body and facial acupuncture on reducing frown lines in women aged 30–59 in Mashhad, Iran.

**Patients/Methods:**

In this double‐arm randomized wait‐list controlled trial, 72 participants were randomly assigned to either an intervention group, receiving facial and body acupuncture twice weekly for 6 weeks, or a control group with no treatment. The primary outcome was assessed using the Global Aesthetic Improvement Scale (GAIS) based on standardized photographs. Secondary outcomes included the Subject Satisfaction Scale (SSS) and Quality of Life (QOL) scores. Measurements were taken at three time points: week 0 (pre‐treatment), week 7 (post‐treatment), and week 12 (follow‐up).

**Results:**

At week 7, 63% of the intervention group showed reduced frown lines at rest, and 72% during maximum frowning, significantly outperforming the control group. The improvements observed in the intervention group persisted at week 12 with 68.6% at rest and 57.2% at maximum frown. The SSS indicated that 72.2% and 62.9% of the intervention group were satisfied with their frown lines at weeks 7 and 12, respectively. Notable QOL improvements in social functioning were observed in the intervention group compared to the control group at both weeks 7 and 12. No serious adverse effects were reported; minor bleeding occurred in 4.86% of treatment sessions, resulting in bruising in 0.69%.

**Conclusion:**

This study demonstrates that facial and body acupuncture is an effective and safe method for reducing frown lines.

**Trial Registration:**

IRCT20230204057316N1 (https://irct.behdasht.gov.ir/trial/68408)

## 1 Introduction

Facial rejuvenation has become one of the most popular voluntary medical interventions, not only because of the growing number of elderly individuals seeking such treatments due to increased life expectancy [1], but also due to the societal emphasis on the importance of physical appearance in contemporary culture [2]. A recent blinded online survey conducted by the American Society of Dermatological Surgeons in 2023 revealed that 70% of individuals are contemplating a cosmetic procedure [3]. A young appearance has emerged as a representation of financial prosperity, social approval, and personal contentment [2]. It is believed that facial rejuvenation can lead to positive changes in feelings of femininity and attractiveness and improve social skills in women, beyond looking younger [4]. Youthfulness and skin health are important factors in the perception of quality of life (QOL) [5].

A stressful lifestyle that requires frequent frowning in the glabellar area has made frown lines the most common wrinkle in the upper third of the face. The glabellar lines can be vertical, horizontal, or oblique, contingent upon the predominant activity of specific facial muscles, namely the corrugator supercilii, procerus, or a combination of both [6].

While invasive facial rejuvenation techniques, including cosmetic surgery, can effectively enhance a youthful look, they do not address the fundamental factors contributing to facial aging. Furthermore, similar to other surgical interventions, these procedures can be costly and carry inherent risks [7]. Consequently, minimally invasive techniques, such as botulinum toxin injections (Botox), are frequently employed as alternatives to surgical procedures due to their lower cost and fewer complications, along with relatively rapid effects. However, Botox can lead to unpleasant, long‐term, and difficult‐to‐treat side effects, including eyebrow ptosis, facial asymmetry, and expressionless faces [8]. To mitigate these adverse effects, simpler alternative approaches to facial rejuvenation, such as acupuncture, have gradually gained popularity [9, 10]. Traditional Chinese Medicine (TCM) adopts a holistic approach that simultaneously addresses the entire body while targeting specific ailments or localized areas. Through acupuncture, a modality of TCM, facial rejuvenation is achieved by promoting overall health and wellness. Acupuncture posits that the condition of the face reflects the health of the internal organs [10]. The risk of serious, unpleasant, long‐lasting, and difficult‐to‐treat side effects associated with acupuncture is minimal, leading Vincent (2001) to conclude that acupuncture is safe in experienced hands [11].

Acupuncture can treat facial wrinkles, such as frown lines, by using distal points (body acupuncture) to influence the overall health of the skin, in addition to local and adjacent points (facial acupuncture), which are used to improve the specific wrinkles. Unfortunately, studies conducted in the field of aesthetic acupuncture are scarce, often consisting of single‐arm studies that focus on wrinkles in the lower two‐thirds of the face and primarily utilize facial points [5, 10, 12, 13]. This study aimed to evaluate the effectiveness of combined facial and body acupuncture on glabellar lines through a randomized controlled trial for the first time.

## 2 Materials and Methods

This single‐center, parallel, randomized waitlist‐controlled trial evaluated the effectiveness of face and body acupuncture treatment (intervention group) compared to a waitlist (control group) among women aged 30 to 59 years who exhibit glabellar frown lines. The study was conducted at the acupuncture clinic of Imam Reza Hospital, Mashhad University of Medical Sciences, Mashad Iran. Detailed information regarding the sample size, recruitment process, and criteria for inclusion and exclusion has been previously outlined in the study protocol article [14]. Participants were enrolled after evaluating their eligibility, as well as obtaining their informed consent, which included permission for the publication of participant photographs. To facilitate random allocation, a permutation block method was employed to create the allocation sequence, utilizing the sealedenvelope website (http://www.sealedenvelope.com). Further details regarding the random allocation process can be found in the study protocol article [14]. In the current study, it was not possible to blind either the patients or the acupuncturist. Nevertheless, the outcome assessors, who were outside the research team, were unaware of the group assigned to the participants. The data analyzer was kept blind to the group allocation of each participant by utilizing A and B codes in the datasheet.

**FIGURE 1:**
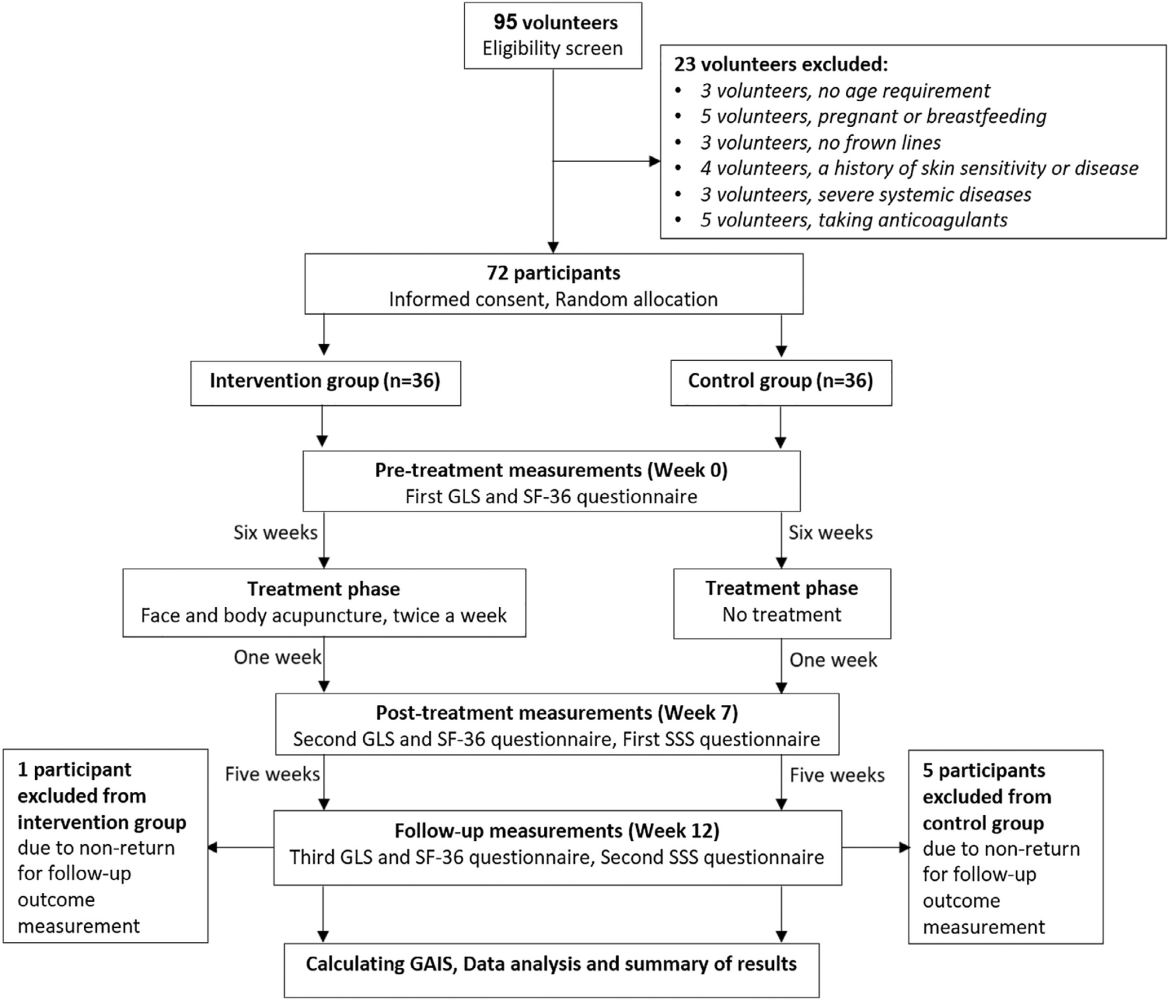
Study flowchart including timeline of enrollment, interventions, and assessments.

The 72 eligible volunteers were randomly assigned to two groups of 36, the intervention group and the control group, in a 1:1 ratio. Figure 1 presents a timeline of participant involvement and a flowchart outlining the study procedures. The study has been officially registered with the Iranian Registry of Clinical Trials under the registration number IRCT20230204057316N1 (https://irct.behdasht.gov.ir/trial/68408).

### 2.1 Interventions

#### 2.1.1 Intervention Group

The acupuncture treatment protocol for the intervention group was documented in compliance with the Standards for Reporting Interventions in Clinical Trials of Acupuncture (STRICTA). The selection of acupuncture points was based on customized Western techniques, employing a list of points that have been previously documented as effective for facial rejuvenation [15] and achieving a consensus informed by our own expertise.

The acupuncture points utilized in body acupuncture were LR3 (Taichong), ST36 (Zusanli), SP9 (Yinlingquan), SP10 (Xuehai), LI4 (Hegu), LU7 (Lieque), PC6 (Neiguan), LI11 (Quchi), and DU20 (Baihui). Needling was performed bilaterally using 25 × 0.25 mm needles, with a total of 17 needles employed throughout the body. Points used for face acupuncture included BL2 (Zanzhu), Ex‐HN4 (Yuyao), TB23 (Sizhukong), and Ex‐HN3 (Yintang), which were needled bilaterally with 13 × 0.18 mm needles, totaling 7 needles for the face. The depth of needle insertion was determined according to established acupuncture guidelines. The acupuncture points were consistent for all participants, regardless of their syndrome differentiation, and all points were needled with the even method. Ultimately, intradermal needles measuring 5 × 0.22 mm were used at the depth of the frown line, spaced 2 mm apart, in an upward direction and at a 45° angle to the skin. All needles were manufactured by Huanqiu (China) and were maintained in position for 20 min during each treatment session before removal. In the event of bleeding following needle removal, the site was compressed with cotton wool for 1 min to reduce the risk of additional bruising. Should bruising develop, Arnica ointment was applied. Acupuncture treatments were administered biweekly for 6 weeks (a total of 12 sessions) by a skilled acupuncturist [HA] while the participant was lying supine.

#### 2.1.2 Control Group

The control group underwent a 6‐week observation period without any intervention, remaining on a waiting list. At the conclusion of the study, this group was offered complimentary acupuncture treatment.

### 2.2 Outcome Measurements

#### 2.2.1 Primary Outcome

**FIGURE 2:**
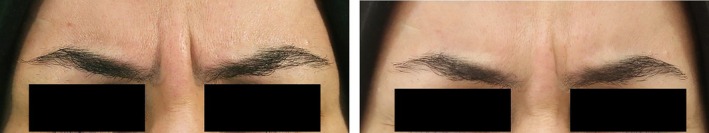
A photograph illustrating much‐improved frown lines under maximum frown condition. The image on the left was captured during the pre‐treatment phase, showing a GLS mean of 2.5 for two frown lines. In contrast, the image on the right was taken during the follow‐up phase, reflecting a GLS mean of 0.5 for the same two frown lines.

Participants from both groups were photographed at rest and during maximum frowning using a Canon 5D Mark III digital camera (Japan) at three time points: pre‐treatment (week 0), post‐treatment (week 7), and follow‐up (week 12). The lighting and other photographic parameters were standardized according to the guidelines established in a previously published protocol article [14]. The Glabellar Line Scale (GLS) was assessed using photographs taken of each participant at rest and during maximum frowning at each time point by three trained independent physicians from the research team. The mean score from these assessors was recorded as the GLS for each case. Each frown line was rated on a 4‐point scale: 0 = no line, 1 = mild, 2 = moderate, and 3 = severe. In instances where participants exhibited more than one frown line, the average score was calculated (Figure 2).

Global Aesthetic Improvement Scale (GAIS) evaluated the extent of improvement in frown lines by utilizing GLS at weeks 7 and 12, comparing the results to week 0 for each volunteer. The GAIS employs a five‐point rating system: (+2) much improved, indicating an enhancement of at least two degrees or achieving a zero‐degree (absence of frown lines); (+1) improved, less than two degrees of improvement without reaching zero; (0) no change; (−1) worse, less than two degrees of worsening; and (−2) much worse, denoting a decline of at least two degrees.

#### 2.2.2 Secondary Outcomes

The Subject Satisfaction Scale (SSS) was administered to participants in both groups during weeks 7 and 12. Self‐evaluation using the SSS consists of five levels: +2 = very satisfied, +1 = satisfied, 0 = no difference, −1 = unsatisfied, and −2 = very unsatisfied.

The QOL score was determined using the SF‐36 version 2 questionnaire. This assessment was conducted in both groups at weeks 0, 7, and 12. To calculate the QOL score, the cumulative scores of the questions related to each of the eight subscales were divided by the total number of questions within that subscale, resulting in an average score ranging from 0 to 100. Subsequently, the average score for each subscale at week 0 was subtracted from the average score of that subscale at weeks 7 or 12. This calculation yielded the difference in the score of that subscale during the post‐treatment or follow‐up phases compared to the pre‐treatment phase. A positive numerical difference in the score of a subscale indicates an improvement in that subscale, while a negative difference suggests a decline.

### 2.3 Statistical Analysis

The analysis utilized SPSS (Version 16.0) to report descriptive statistics for both qualitative and quantitative variables, including frequency, percentage, mean, standard deviation, median, and quartiles. A t‐test was employed for comparing normally distributed quantitative data between two groups, while the Mann–Whitney test was applied to non‐normally distributed data. ANOVA and the Kruskal‐Wallis test were utilized for comparisons involving more than two groups. Qualitative variables were analyzed using the Chi‐square and Fisher's exact tests. Paired t‐tests and Wilcoxon tests were conducted for before‐and‐after comparisons. The intention‐to‐treat analysis addressed missing data, and regression models were employed to correct for confounding factors. All tests were two‐sided, with a significance level of *p* < 0.05.

## 3 Results

As indicated in Table 1, the demographic analysis revealed that age was the only variable showing a significant difference between the two participant groups, suggesting that the control group was slightly younger than the intervention group (*p* = 0.03).

**TABLE 1:** Demographic information of participants.

Variable	Intervention Group (*n* = 36)	Control Group (*n* = 36)	*p*
Age (years) *Mean ± SD*	46.89 ± 7.13	42.97 ± 8.04	0.03
Height (cm) *Mean ± SD*	160.75 ± 5.46	162.28 ± 5.73	0.2
Weight (kg) *Mean ± SD*	66.67 ± 10.30	66.58 ± 10.38	0.9
*Education n (%)*			
Diploma	6 (16.7)	3 (8.3)	—
Associate	3 (8.3)	2 (5.6)	
Bachelor	12 (33.3)	13 (36.1)	
Master	11 (30.6)	16 (44.4)	
PhD	4 (11.1)	2 (5.6)	
*Occupation n (%)*			
Student	5 (13.9)	6 (16.7)	—
Employee	23 (63.9)	25 (69.4)	
Cleaner	1 (2.8)	1 (2.8)	
Housewife	3 (8.3)	2 (5.6)	
Retired	4 (11.1)	1 (2.8)	
Shopkeeper	0 (0)	1 (2.8)	
*Marital status n (%)*			
Single	7 (19.4)	11 (30.6)	0.4
Married	27 (75)	22 (61.1)	
Divorced/Widow	2 (5.6)	3 (8.3)	
Smoking history *n (%)*	12 (33.3)	11 (30.6)	0.8

*Note:* The initial three variables were analyzed using a paired t‐test, while the remaining variables were assessed using a chi‐square test.

**FIGURE 3:**
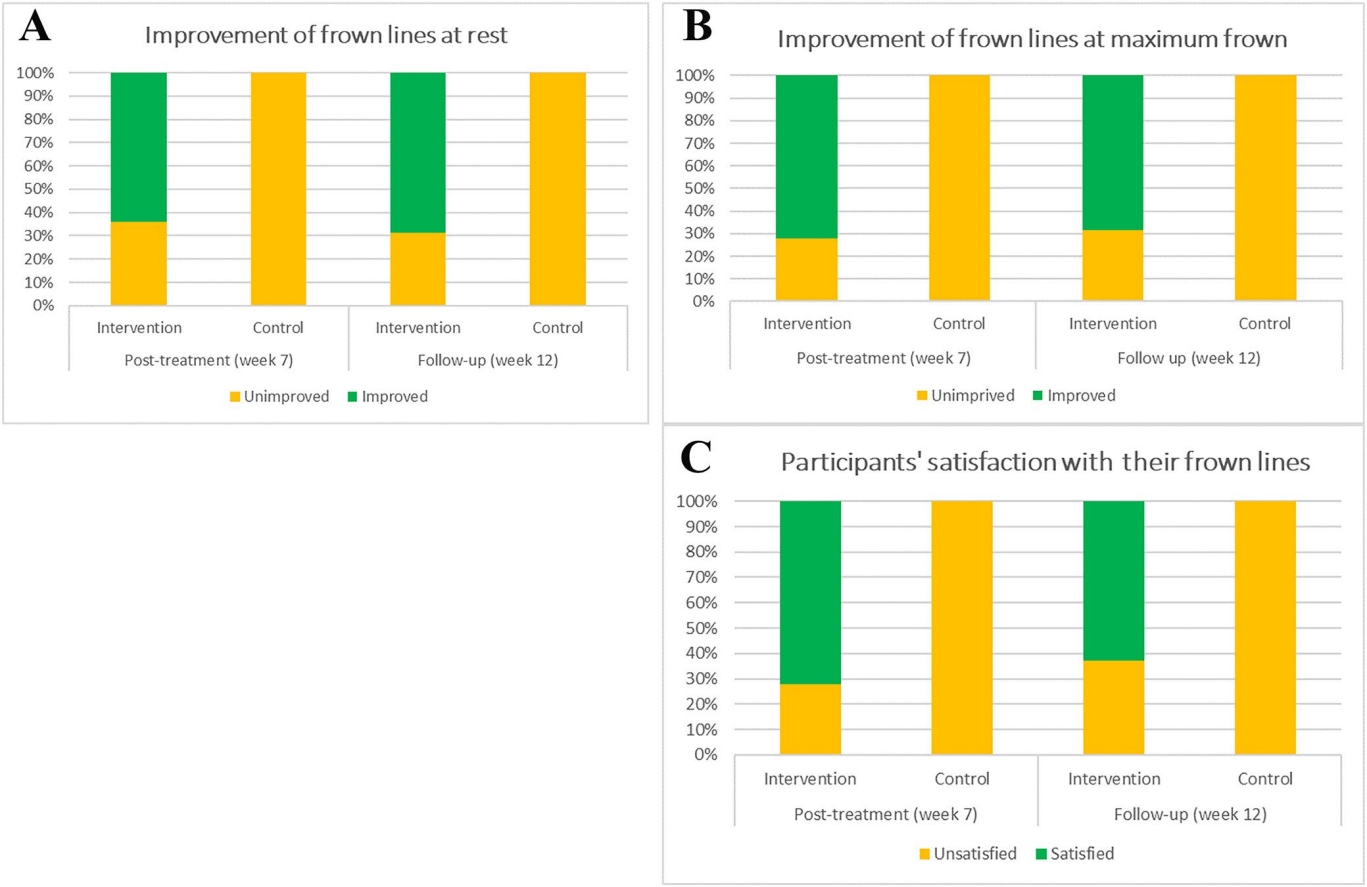
A comparison of frown lines at rest (A) and during maximum frown (B) between improved (regardless of severity) and unimproved (including based on the GAIS scale at weeks 7 (post‐treatment) and 12 (follow‐up), relative to week 0 (pre‐treatment) in both the intervention and control groups. (C) presents participants' opinions regarding their frown line status, categorizing them as satisfied (with any severity) or unsatisfied (which includes “very unsatisfied,” “unsatisfied,” and “no difference”) at weeks 7 and 12, again in comparison to week 0, across both groups.

The improvement in frown lines observed at rest and during maximum frowning, as measured by the GAIS scale at week 7 in the intervention group [median, (first quartile, third quartile)] was 0.5 (0, 1) and 1 (0.125, 1), respectively. This improvement surpassed that of the control group, which exhibited no change, recorded as 0 (0, 0) in both conditions, as determined by the Mann–Whitney test (*p* < 0.0001 for each condition). At week 12, the intervention group demonstrated an improvement in frown lines of 1 (0, 1) at both rest and maximum frowning, again exceeding the control group, which remained at 0 (0, 0) for both scenarios, as indicated by the Mann–Whitney test (*p* < 0.0001 for each). The results regarding the improvement of frown lines, both at rest and during maximum frowning, along with participants' satisfaction levels assessed through the five‐point GAIS and SSS scales at weeks 7 and 12, are presented in Tables 2 and 3, respectively. When categorizing the improvement of frown lines based on the five‐point GAIS scale, two groups emerge: “improved” (encompassing any level of improvement) and “unimproved” (including no change or any degree of worsening). Similarly, participants' satisfaction with their frown lines, as measured by the five‐point SSS scale, can be divided into “satisfied” (covering any level of satisfaction) and “unsatisfied” (including no change or any level of unsatisfaction). This categorization allows for the application of a Fisher's exact test (Figure 3). Notably, no spontaneous improvement in frown lines due to natural processes was observed in the control group at weeks 7 and 12. The Fisher's exact test indicated that, at both weeks 7 and 12, there was a significant increase in participants' satisfaction regarding their frown line status based on the SSS in the intervention group compared to the control group (*p* < 0.0001). Additionally, improvements in frown lines in both conditions (at rest and during maximum frowning) were also observed according to the GAIS (*p* < 0.0001 for each condition).

**TABLE 2:** Global Aesthetic Improvement Scale at rest (GAIS‐R) and at maximum frown (GAIS‐F) in intervention and control groups at post‐treatment (week 7) and follow‐up (week 12) assessments.

Assessment time		Post‐treatment phase (week 7)		Follow‐up phase (week 12)	
Group		Intervention	Control	Intervention	Control
GAIS		(*n* = 36)	(*n* = 36)	(*n* = 35)	(*n* = 31)
GAIS‐R	*Much improved, n (%)*	11 (30.6)	0 (0)	15 (42.9)	0 (0)
	*Improved, n (%)*	12 (33.3)	0 (0)	9 (25.7)	0 (0)
	*No change, n (%)*	13 (36.1)	36 (100)	11 (31.4)	30 (96.8)
	*Worse, n (%)*	0 (0)	0 (0)	0 (0)	1 (3.2)
	*Much worse, n (%)*	0 (0)	0 (0)	0 (0)	0 (0)
GAIS‐F	*Much improved, n (%)*	4 (11.1)	0 (0)	5 (14.3)	0 (0)
	*Improved, n (%)*	22 (61.1)	0 (0)	15 (42.9)	0 (0)
	*No change, n (%)*	10 (27.8)	32 (88.9)	15 (42.9)	25 (80.7)
	*Worse, n (%)*	0 (0)	4 (11.1)	0 (0)	5 (16.1)
	*Much worse, n (%)*	0 (0)	0 (0)	0 (0)	1 (3.2)

**TABLE 3:** Subject Satisfaction Scale (SSS) for intervention and control groups at post‐treatment (week 7) and follow‐up (week 12) assessments.

Assessment time	Post‐treatment phase (week 7)		Follow‐up phase (week 12)	
Group	Intervention	Control	Intervention	Control
SSS	(*n* = 36)	(*n* = 36)	(*n* = 35)	(*n* = 31)
Very satisfied, *n* (%)	4 (11.1)	0 (0)	3 (8.6)	0 (0)
Satisfied, *n* (%)	22 (61.1)	0 (0)	19 (54.3)	0 (0)
No difference, *n* (%)	10 (27.8)	32 (88.9)	13 (37.1)	27 (87.1)
Unsatisfied, *n* (%)	0 (0)	4 (11.1)	0 (0)	2 (6.5)
Very unsatisfied, *n* (%)	0 (0)	0 (0)	0 (0)	2 (6.5)

According to the SF‐36 questionnaire, the overall QOL score in the intervention group at week 7 showed a median difference of 2.9, with a first quartile of −3.8 and a third quartile of 8.7, compared to week 0. This improvement was notable when contrasted with the control group, which exhibited a score of −0.35 (−7.5, 3.5), as determined by the Mann–Whitney test (*p* = 0.03). Furthermore, when comparing the post‐treatment phase to the pre‐treatment phase, the intervention group demonstrated a significant increase in the social functioning subscale score of 0 (0, 21.9) versus 0 (−21.9, 9.4) (*p* = 0.03), a pain score of 1.2 (0, 12.5) compared to 0 (−12.5, 0) (*p* = 0.004), and role limitations due to physical health with a score of 0 (0, 25) against 0 (−25, 0) (*p* = 0.001) when compared to the control group. With the exception of the social functioning subscale, which indicated a borderline significant increase in the intervention group at week 12 with a score of 0 (0, 12.5) compared to the control group's −12.5 (−12.5, 12.5) (*p* = 0.05), no significant differences in QOL scores were observed between the two groups during the follow‐up phase relative to the pre‐treatment phase.

No serious adverse events, infections, or localized reactions were observed. Out of 432 treatment sessions, minor bleeding from the needle exit site occurred in 21 sessions (4.86%), resulting in bruising (ecchymosis) in only three instances (0.69%). These bruises resolved quickly within a few days with the application of arnica ointment. Not a single case of treatment discontinuation due to side effects was reported among the participants in the intervention group.

## 4 Discussion

In this study, which to our knowledge represents the inaugural double‐arm clinical trial to investigate the effect of acupuncture on glabellar lines, the intervention group received face and body acupuncture in conjunction with intradermal needling along the frown line, while the control group was monitored without any intervention for 6 weeks.

The volunteers in the control group were slightly younger than those in the intervention group. Given that a younger age may be advantageous in the context of improving facial wrinkles, this disparity in the current study may be seen as beneficial to the control group in comparison to the intervention group.

The study found that about two‐thirds of participants in the intervention group expressed satisfaction with their frown lines by week 7, maintaining this level of satisfaction until week 12. This finding aligns with results from a recent retrospective study examining the effectiveness of acupuncture on nasolabial and marionette lines [16]. Additionally, an analysis conducted by Dayan et al. (2022) revealed that over 90% of patients experienced improvement in frown lines following Botox at week 4; however, satisfaction levels decreased to 70% by week 36 [17]. While Botox delivers rapid and significant results, it may result in an unnatural, expressionless face, which may not only be short‐term due to the paralysis of facial muscles but can also be long‐term due to alterations in neural activity related to emotional processing in the brain [18]. Conversely, acupuncture, as will be discussed later, not only alleviates spasms in the facial muscles without causing paralysis but also induces beneficial changes throughout the body that enhance vitality and promote a refreshed appearance, contributing to a naturally youthful visage.

The study revealed a significant improvement in total QOL score within the intervention group at week 7, primarily due to improvements in pain and role limitations due to physical health subscales (physical health indicators), with some enhancement in the social functioning subscale. However, at week 12, physical health indicators in the intervention group were not significantly different from the control group, although the social functioning subscale score remained significantly higher. The pain reduction may be attributed to needling at specific acupoints known for pain relief, such as LR3, ST36, LI4, and LI11; nonetheless, some experts argue that the analgesic effects could be essentially non‐specific. Pain alleviation may also help reduce frown lines by relaxing the muscles involved. The findings related to pain reduction in this study were limited to temporary effects, as it did not specifically target participants' pain conditions. Additionally, improvements in social functioning may stem from increased self‐confidence and satisfaction with aesthetic outcomes, which persisted during follow‐up assessments.

The study reported significant improvement in both resting and maximum frown lines among participants in the intervention group. At week 7, 63.9% of participants showed improvement at rest, increasing to 68.6% by week 12, while 72.2% improved at maximum frown at week 7, dropping to 57.2% by week 12. In contrast, the control group showed no improvements. Acupuncture was more effective in reducing frown lines during maximum frown than that of resting state at week 7. The study suggested that pain heightened muscle activity responsible for frowning, leading to dynamic frown lines (frown lines during maximum frown). As the analgesic effects of acupuncture waned by week 12, participants experienced a reduction in dynamic frown lines, while static frown lines (frown lines at rest) slightly increased by week 12 due to the time‐consuming process of collagen synthesis.

During the treatment sessions and in the post‐treatment and follow‐up assessments, no major or dangerous side effects, infections, or localized reactions were observed. Minor bleeding occurred in 4.86% of sessions, with only three cases (0.69%) resulting in bruising that resolved quickly with arnica ointment.

While no direct studies on acupuncture's effect on frown lines were found, the results align with existing research on mid‐ and lower‐face rejuvenation through acupuncture [5, 10, 12, 13, 16, 19]. In comparison, a review of “botulinum toxin type A” injections indicated that 67.5% to 70.4% of participants experienced significant improvements in frown lines after 30, which is comparable to the improvement rates observed in our study. However, these injections were associated with a complication rate ranging from 0.4% to 1.2% (brow ptosis 0.4%, eyelid ptosis 0.1%–2.8%, blurred vision 0.4%, and diplopia 0.4%). In addition, a one‐year study of patients who underwent Botox reported a significant incidence of systemic adverse events, including cardiac, ear and labyrinth, ocular, gastrointestinal and hepatic, immune, hematological and lymphatic, and endocrine complications [20].

Intradermal needles and specific facial acupuncture points (BL2, TB23, Ex‐HN3, and Ex‐HN4) play a significant role in improving frown lines through mechanisms explained by conventional Western medicine. Insertion of intradermal needles into frown lines induces micro‐injuries that stimulate the release of growth factors and activate fibroblasts, which play a critical role in collagen production. This cascade of events promotes collagen deposition and reorganization, leading to a gradual reduction in the appearance of frown lines over time [8, 9, 10, 15]. This process also activates local nerves, leading to the release of neuropeptides that cause vasodilation and increased blood circulation, a phenomenon known as the “axonal reflex” [21]. The hyperemia observed at the site of intradermal needle insertion due to “axonal reflex” can help the frown line to repair. Repeated contraction, spasm, and shortening of the muscles of facial expression lead to the appearance of dynamic wrinkles on the face [22]. The insertion of acupuncture needles into the contracted muscles, including the origin, insertion, and belly of the Corrugator supercilii muscle (BL2, TB23, and Ex‐HN4) as well as the Procerus muscle (Ex‐HN3), which are associated with frowning, facilitates the release of muscle spasms and alleviates the shortening of these muscles, thereby reducing the appearance of frown lines [5, 23]. Moisturizing the skin is crucial for anti‐aging, with water and lipid balance in the stratum corneum being essential for maintaining skin moisture. Stimulating some acupuncture points on the body can play an important role in controlling skin pores, sweating, oil content, and skin moisture by activating the somato‐autonomic reflex and regulating the autonomic system [24], and thus may reduce skin wrinkles, including frown lines, by improving skin moisture and oiliness. Preliminary results in at least one study confirm that cosmetic acupuncture has been able to increase the water and lipid content of facial skin [12]. Furthermore, acupuncture influences neurotransmitters like endorphins, serotonin, and dopamine in the limbic system, which regulate emotions and muscle use [23]. By modulating neurotransmitters involved in emotions, acupuncture can reduce overuse of the muscles responsible for frowning, thereby helping to improve frown lines.

## 5 Conclusion

This study, the first randomized, double‐arm clinical trial, revealed that facial and body acupuncture not only reduced frown lines in women aged 30 to 59 years, but also increased their satisfaction level and social functioning. However, the results of this study may not be easily generalized due to limitations such as small sample size, lack of racial and gender diversity, and a short follow‐up period. The careful selection of an appropriate control group, along with the blinding of both assessors and the analyst, is considered a notable strength of this study. Based on a cross‐sectional study, if the aim of selecting a control group is to examine the effect of natural progression of the disease (the effect of time), a waiting list would be an appropriate control method [25]. To overcome the limitations identified in the present study, future clinical trials should incorporate larger sample sizes, diverse populations in terms of gender and race, extended follow‐up assessments, and multi‐arm trial designs. These designs should include no‐treatment, body acupuncture, facial acupuncture, and combined body and facial acupuncture groups to elucidate the differential effects of these interventions on frown lines. Further research could also focus on comparing the effectiveness and safety of acupuncture against other treatments for frown lines.

## Author Contributions

H.H.: conceptualization, project administration, writing – original draft preparation. M.J.Y.: conceptualization, resources, writing – reviewing and editing. S.K.F.: conceptualization, writing – reviewing and editing. M.K‐R.: methodology, writing – reviewing and editing. H.A.: conceptualization, supervision, funding acquisition, investigation, writing – reviewing and editing. All authors read and approved the final version.

## Ethics Statement

This clinical trial was in accordance with the principles of the Declaration of Helsinki and was granted on 7 April 2023 by the Ethics Committee of Mashhad University of Medical Sciences (Ethics Reference No: IR.MUMS.REC.1402.011, https://ethics.research.ac.ir/ProposalCertificateEn.php?id=326015&Print=true&NoPrintHeader=true&NoPrintFooter=true&NoPrintPageBorder=true&LetterPrint=true).

## Consent

All participants provided informed consent, which encompassed authorization for the publication of their photographs.

## Conflicts of Interest

The authors declare no conflicts of interest.

## Data Availability

The data that support the findings of this study are available from the corresponding author upon reasonable request.

## References

[1] M. V. Hînganu , R. P. Cucu , and D. Hînganu , “Personalized Research on the Aging Face—A Narrative History,” Journal of Personalized Medicine 14, no. 4 (2024): 343. 38672970 10.3390/jpm14040343 PMC11050910

[2] M. A. Gupta and B. A. Gilchrest , “Psychosocial Aspects of Aging Skin,” Dermatologic Clinics 23, no. 4 (2005): 643–648. 16112440 10.1016/j.det.2005.05.012

[3] “ASDS Consumer Survey on Cosmetic Dermatologic Procedures 2023,”, https://www.asds.net/portals/0/PDF/consumer‐survey‐2023‐infographic.pdf.

[4] M. J. Reilly , J. A. Tomsic , S. J. Fernandez , and S. P. Davison , “Effect of Facial Rejuvenation Surgery on Perceived Attractiveness, Femininity, and Personality,” JAMA Facial Plast Surg 17, no. 3 (2015): 202–207. 25856281 10.1001/jamafacial.2015.0158

[5] Y. Yun , S. Kim , M. Kim , K. Kim , J.‐S. Park , and I. Choi , “Effect of Facial Cosmetic Acupuncture on Facial Elasticity: An Open‐Label, Single‐Arm Pilot Study,” Evidence‐Based Complementary and Alternative Medicine 2013, no. 1 (2013): 424313. 23983778 10.1155/2013/424313 PMC3745857

[6] O. Friedman , “Changes Associated With the Aging Face,” Facial Plastic Surgery Clinics of North America 13, no. 3 (2005): 371–380. 16085282 10.1016/j.fsc.2005.04.004

[7] R. Honigman and D. J. Castle , “Aging and Cosmetic Enhancement,” Clinical Interventions in Aging 1, no. 2 (2006): 115–119. 18044108 10.2147/ciia.2006.1.2.115 PMC2695163

[8] M. Kassir , M. Gupta , H. Galadari , et al., “Complications of Botulinum Toxin and Fillers: A Narrative Review,” Journal of Cosmetic Dermatology 19, no. 3 (2020): 570–573. 31889407 10.1111/jocd.13266

[9] V. C. Doran , “An Introduction to Facial Revitalisation Acupuncture,” European Journal of Oriental Medicine 5, no. 5 (2007): 4.

[10] J. B. Barrett , “Acupuncture and facial rejuvenation,” Aesthetic Surgery Journal 25, no. 4 (2005): 419–424. 19338843 10.1016/j.asj.2005.05.001

[11] C. Vincent , “The Safety of Acupuncture: Acupuncture Is Safe in the Hands of Competent Practitioners,” British Medical Journal Publishing Group 323, no. 7311 (2001): 467–468. 10.1136/bmj.323.7311.467 PMC1121068 11532826

[12] N. Donoyama , A. Kojima , S. Suoh , and N. Ohkoshi , “Cosmetic Acupuncture to Enhance Facial Skin Appearance: A Preliminary Study,” Acupuncture in Medicine 30, no. 2 (2012): 152–153. 22534726 10.1136/acupmed-2012-010156

[13] Y. Yun and I. Choi , “Effect of Thread Embedding Acupuncture for Facial Wrinkles and Laxity: A Single‐Arm, Prospective, Open‐Label Study,” Integrative Medicine Research 6, no. 4 (2017): 418–426. 29296569 10.1016/j.imr.2017.09.002 PMC5741386

[14] H. Haghir , M. J. Yazdanpanah , M. Khadem‐Rezaiyan , F. Bidouei , and H. Azizi , “Effects of Face and Body Acupuncture on Glabellar Frown Lines in Women Aged 30‐59: A Study Protocol for a Double‐Arm Randomized Waitlist‐Controlled Trial,” Journal of Acupuncture and Meridian Studies 17, no. 6 (2024): 1–8. 39722645 10.51507/j.jams.2024.17.6.221

[15] P. Adkins , Facial Enhancement Acupuncture: Clinical Use and Application (Singing Dragon, 2013).

[16] H. Cheng , L. Xie , T. Wang , and B. Shi , “Effectiveness of Acupuncture Therapy on Improvement of Nasolabial Folds and Marionette Lines: A Retrospective Study,” Health Science Reports 7, no. 8 (2024): e70014. 39175601 10.1002/hsr2.70014 PMC11339128

[17] S. Dayan , J. Joseph , A. Moradi , et al., “Subject Satisfaction and Psychological Well‐Being With Escalating abobotulinumtoxinA Injection Dose for the Treatment of Moderate to Severe Glabellar Lines,” Journal of Cosmetic Dermatology 21, no. 6 (2022): 2407–2416. 35266281 10.1111/jocd.14906 PMC9322427

[18] S. Stark , C. Stark , B. Wong , and M. F. Brin , “Modulation of Amygdala Activity for Emotional Faces due to Botulinum Toxin Type A Injections That Prevent Frowning,” Scientific Reports 13, no. 1 (2023): 3333. 36849797 10.1038/s41598-023-29280-x PMC9971043

[19] J. H. Cho , H. J. Lee , K. J. Chung , B. C. Park , M. S. Chang , and S. K. Park , “Effects of Jae‐Seng Acupuncture Treatment on the Improvement of Nasolabial Folds and Eye Wrinkles,” Evidence‐Based Complementary and Alternative Medicine 2015, no. 1 (2015): 273909. 26064158 10.1155/2015/273909 PMC4434186

[20] M. B. Gadarowski , R. I. Ghamrawi , S. L. Taylor , and S. R. Feldman , “PrabotulinumtoxinA‐Xvfs for the Treatment of Moderate‐To‐Severe Glabellar Lines,” Annals of Pharmacotherapy 55, no. 3 (2021): 354–361. 32698599 10.1177/1060028020943527

[21] A. White , M. Cummings , and J. Filshie , An Introduction to Western Medical Acupuncture (Elsevier Health Sciences, 2018).

[22] J. P. Farkas , J. E. Pessa , B. Hubbard , and R. J. Rohrich , “The Science and Theory Behind Facial Aging,” Plastic and Reconstructive Surgery. Global Open 1, no. 1 (2013): e8–e15. 25289202 10.1097/GOX.0b013e31828ed1da PMC4174174

[23] K. J. Cheng , “Neurobiological Mechanisms of Acupuncture for Some Common Illnesses: A clinician's Perspective,” Journal of Acupuncture and Meridian Studies 7, no. 3 (2014): 105–114. 24929454 10.1016/j.jams.2013.07.008

[24] K. Kimura , K. Masuda , and I. Wakayama , “Changes in Skin Blood Flow and Skin Sympathetic Nerve Activity in Response to Manual Acupuncture Stimulation in Humans,” American Journal of Chinese Medicine 34, no. 2 (2006): 189–196. 16552831 10.1142/S0192415X06003758

[25] J. Liu , L. Li , X. Luo , et al., “Specification of Interventions and Selection of Controls in Randomized Controlled Trials of Acupuncture: A Cross‐Sectional Survey,” Acupuncture in Medicine 40, no. 6 (2022): 524–537. 36039602 10.1177/09645284221117848

